# Identification of *cpxS* mutational resistome in *Pseudomonas aeruginosa*

**DOI:** 10.1128/aac.00921-23

**Published:** 2023-10-06

**Authors:** Zhe-Xian Tian, Yi-Ping Wang

**Affiliations:** 1 State Key Laboratory of Protein and Plant Gene Research,School of Life Sciences, Peking University, Beijing, China; Shionogi Inc., Florham Park, New Jersey, USA

**Keywords:** CpxS, mutational resistome, CpxR, MexAB-OprM, *Pseudomonas aeruginosa*

## Abstract

*Pseudomonas aeruginosa* easily produces drug-resistant mutants. A large number of mutational resistome genes exist in the genome of *P. aeruginosa*. In this study, whole genome sequencing analysis of a multidrug-resistant *P. aeruginosa* strain isolated by *in vitro* antibiotic treatment showed a mutation in the *cpxS* gene. Random mutagenesis of *cpxS* was conducted and introduced into the PA14Δ*cpxS* strain. Numerous CpxS mutants, including 14 different single amino acid substitutions, were identified, which led to reduced antibiotic susceptibility. Moreover, some of them were also present in the published genomes of *P. aeruginosa* isolates. Around *cpxS*, a gene coding for a putative sensor kinase, the nearest gene coding for a response regulator is *cpxR* in the genome of *P. aeruginosa*. Deletion of *cpxR* restored antibiotic susceptibility in the above *cpxS* mutant strains. As an extension of our previous work, where the expression of the *mexAB-oprM* operon is directly activated by CpxR in *P. aeruginosa*, in this study, we showed that the expression of the *mexA* promoter was increased in the above *cpxS* mutant strains in a *cpxR*-dependent manner, and *mexA* is prerequisite for the reduced antibiotic susceptibility. Therefore, we propose that the putative sensor kinase CpxS, together with CpxR, comprises a two-component regulatory system regulating the expression of the *mexAB-oprM* operon in *P. aeruginosa*. Our work indicates that *cpxS*, as a novel member of mutational resistome, plays important roles on the development of multidrug resistance in *P. aeruginosa*.

## INTRODUCTION

*Pseudomonas aeruginosa*, a major pathogen associated with cystic fibrosis, is well known for its intrinsic resistance to a wide range of antimicrobial agents and its ability to develop multidrug resistance following antibiotic therapy (1). A series of mutational resistome analysis have been carried out unraveling a large number of genes previously unrelated to antibiotic resistance, which exert altered antibiotic susceptibility when they were mutated in *P. aeruginosa* (2 – 7). Although the significance of the mutational resistome has been widely understood, the possible complex linkages among these numerous resistome genes discourage further investigation of the mechanisms behind, while apparent difficulties for differentiating relevant mutations from simple natural polymorphisms hinder the knowledge transfer to clinical application (8).

As the predominant multidrug efflux system, the MexAB-OprM efflux pump is largely responsible for intrinsic and acquired multidrug resistance in *P. aeruginosa* (9). The expression of the *mexAB-oprM* operon was modulated directly by two repressors, MexR and NalD, and indirectly by another repressor, NalC. Mutations causing defective forms of MexR, NalC, and NalD lead to overexpression of the *mexAB-oprM* operon and reduce antibiotic susceptibility in *P. aeruginosa* (10 – 12). In particular, mutations in *mexR* are the major genotypes associated with *nalB*-type strains and are often identified among clinical isolates (13). Besides these transcriptional repressors, a response regulator CpxR can bind and activate the promoter of the *mexAB-oprM* operon and contributes to a novel type of multidrug resistance in *P. aeruginosa* (14).

In Gram-negative pathogens, the response regulator CpxR and its cognate membrane integral sensor kinase, CpxA, form a two-component regulatory system involved in cell envelope protection against various stresses such as pH, salinity, heavy metal, and macromolecule misfolding (15 – 19). As a member of the EnvZ sensor kinase family, CpxA detects envelope stresses by it’s periplasmic domain, delivers the signal through it’s HAMP domain (conserved in histidine kinases, adenylyl cyclases, methyl-accepting chemotaxis proteins and phosphatases), autophosphorylates it’s conserved histidine residue of the Dhp domain (dimerization and histidine phosphotransfer), and finally transfers the phosphoryl group to CpxR, which, in turn, functions as a global transcription regulator for the CpxR regulon (19). So far, little is known about Cpx signal transduction in *P. aeruginosa*.

In this work, a putative sensor kinase gene, *cpxS*, was identified as a member of the mutational resistome by whole genome sequencing and random mutagenesis screening. Subsequent genetic analysis revealed that the expression level of the MexAB-OprM efflux pump was increased in these *cpxS* mutant strains in a *cpxR*-dependent manner.

## MATERIALS AND METHODS

### Whole genome sequencing analysis

The genomes of isolated multidrug-resistant *P. aeruginosa* strains were sequenced using an Illumina HiSeq 4000 system (Illumina, San Diego, CA, USA) at the Beijing Genomics Institute (Shenzhen, China). The sequenced reads were assembled using SOAPdenovo v.1.05 software. Using alignment software MUMmer (http://mummer.sourceforge.net/), we aligned each query sequence with the reference sequence (*P. aeruginosa* UCBPP-PA14, NCBI refseq: GCF_000014625.1).

### Antibiotic susceptibility test

The minimal inhibition concentration (MIC) of each antibiotic was determined on Mueller-Hinton agar by the twofold dilution method as previously described (14). Ciprofloxacin and ofloxacin were purchased from Bio Basic Inc. Ceftazidime was purchased from Sigma-Aldrich. Cefsulodin was purchased from TOKU-E (Japan). Aztreonam was purchased from Selleck.

### Deletion strain construction

Generation of gene locus-deleted *P. aeruginosa* strains was conducted using a method described previously (14). Briefly, the gene locus of *P. aeruginosa* strains was replaced with a fragment containing the *FRT* gentamicin-resistance (Gm^R^) cassette by double-crossover homologous recombination. The Gm^R^ marker in the chromosome was removed by introducing plasmid harboring the Flp recombinase gene. Correct deletion in the constructed mutant was verified by PCR using primers that bound to flanking chromosomal regions. All DNA primers used in this study are listed in Table S1.

### Random mutagenesis of *cpxS* by error-prone PCR

The entire region of *cpxS* region was PCR-amplified using the chromosomal DNA of *P. aeruginosa* PA14 strain as the template and cloned into pUC18. Error-prone PCR method was adapted from the literature (20). Column purified PCR products were digested by *Bam*HI and *Xba*I restriction enzymes and were ligated into the corresponding restriction sites of the stable broad host plasmid pBBR1MCS5 (21). The plasmids were electroporated into the *Escherichia coli* donor strain and transferred into *P. aeruginosa* strains by conjugation.

### Nucleotide substitution in the chromosomal *cpxS*

The target mutant *cpxS* gene fragment was obtained by nest-PCR and introduced into the above PA14Δ*cpxS::Gm^R^* strain. Following double-crossover homologous recombination, we selected out and verified strains sensitive to gentamicin for successful introduction of the target mutant *cpxS* by sequencing.

### β-galactosidase assay for promoter::*lacZ* reporter gene fusion

The *mexA*p::*lacZ* and *cpxP*p::*lacZ* reporter gene fusion plasmids (14) were introduced into *P. aeruginosa* strains by conjugal transfer. Cells were grown overnight in Mueller-Hinton broth (Oxoid) supplemented with appropriate antibiotics, after which they were diluted 1:50 in 5 mL of fresh medium in 50-mL culture flasks at 37°C with mixing at 150 rpm. Cells were recovered during the logarithmic growth phase (OD_600_ = 0.5–0.9). β-galactosidase assays were performed as described previously (14). The results were presented as the mean values from triplicate samples. Statistical analysis was performed using an unpaired two-tailed Student *t*-test.

## RESULTS

### Existence of *cpxS* mutational resistome in *P. aeruginosa*

Previously, we noticed that *in vitro* treatment of lethal levels of ofloxacin and cefsulodin could isolate multidrug-resistant mutants from *P. aeruginosa* PA14 (14). In order to identify the relevant mutational resistome gene, whole genome sequencing analysis was carried out in these strains. One isolate, PA14OCR109, which showed reduced susceptibility to fluoroquinolones and β-lactams (Table 1), had a ^76^CTG → CCG mutation in the position of the 26th residue, causing leucine to proline substitution in the locus of *PA14_22730*, which has been annotated as *cpxS* (22). Deletions of *cpxS* abolished the elevation of the MICs of antibiotics in the PA14OCR109 strain (Table 1).

**TABLE 1:** Minimal inhibitory concentrations of antibiotics in various *cpxS* mutant PA14 strains

Strain	Plasmid	MIC (μg/mL)^*a*^				
		CIP	CAZ	OFX	CFS	ATM
PA14	Null	0.13	1.0	0.5	1.0	4.0
PA14OCR109	Null	0.5	4.0	2.0	4.0	16
PA14OCR109Δ*cpxS*	Null	0.13	1.0	0.5	1.0	4.0
PA14Δ*cpxS*	pVector	0.13	1.0	0.5	1.0	4.0
PA14Δ*cpxS*	pCpxS	0.13	1.0	0.5	1.0	4.0
PA14Δ*cpxS*	pCpxS^L17P^	0.25	2.0	1.0	2.0	8.0
PA14Δ*cpxS*	pCpxS^A20V^	0.25	2.0	1.0	2.0	8.0
PA14Δ*cpxS*	pCpxS^L22P^	0.5	4.0	2.0	4.0	16
PA14Δ*cpxS*	pCpxS^L25P^	0.5	4.0	2.0	4.0	16
PA14Δ*cpxS*	pCpxS^L26P^	0.5	4.0	2.0	4.0	16
PA14Δ*cpxS*	pCpxS^R106C^	0.25	2.0	1.0	2.0	8.0
PA14Δ*cpxS*	pCpxS^L171P^	0.25	2.0	1.0	2.0	8.0
PA14Δ*cpxS*	pCpxS^A201V^	0.25	2.0	1.0	2.0	8.0
PA14Δ*cpxS*	pCpxS^R203H^	0.25	2.0	1.0	2.0	8.0
PA14Δ*cpxS*	pCpxS^S236P^	0.5	4.0	2.0	4.0	16
PA14Δ*cpxS*	pCpxS^S241P^	0.5	4.0	2.0	4.0	16
PA14Δ*cpxS*	pCpxS^A244V^	0.25	2.0	1.0	2.0	8.0
PA14Δ*cpxS*	pCpxS^I279S^	0.25	2.0	1.0	2.0	8.0
PA14Δ*cpxS*	pCpxS^L437P^	0.25	2.0	1.0	2.0	8.0
PA14::*cpxS^S26P^*	Null	0.5	4.0	2.0	4.0	16
PA14::*cpxS^S236P^*	Null	0.5	4.0	2.0	4.0	16
PA14::*cpxS^S241P^*	Null	0.5	4.0	2.0	4.0	16
PA14::*cpxS^S26P^* Δ*cpxR*	Null	0.13	1.0	0.5	1.0	4.0
PA14::*cpxS^S236P^* Δ*cpxR*	Null	0.13	1.0	0.5	1.0	4.0
PA14::*cpxS^S241P^* Δ*cpxR*	Null	0.13	1.0	0.5	1.0	4.0
PA14::*cpxS^S241P^* Δ*cpxR*	pCpxS^S241P^	0.13	1.0	0.5	1.0	4.0
PA14::*cpxS^S26P^* Δ*mexA*	Null	0.06	0.5	0.13	0.5	1.0
PA14::*cpxS^S236P^* Δ*mexA*	Null	0.06	0.5	0.13	0.5	1.0
PA14::*cpxS^S241P^* Δ*mexA*	Null	0.06	0.5	0.13	0.5	1.0
PA14Δ*mexA*	Null	0.06	0.5	0.13	0.5	1.0

^*a*^ CIP, ciprofloxacin; CAZ, ceftazidime; OFX, ofloxacin; CFS, cefsulodin; ATM, aztreonam.

**FIG 1:**
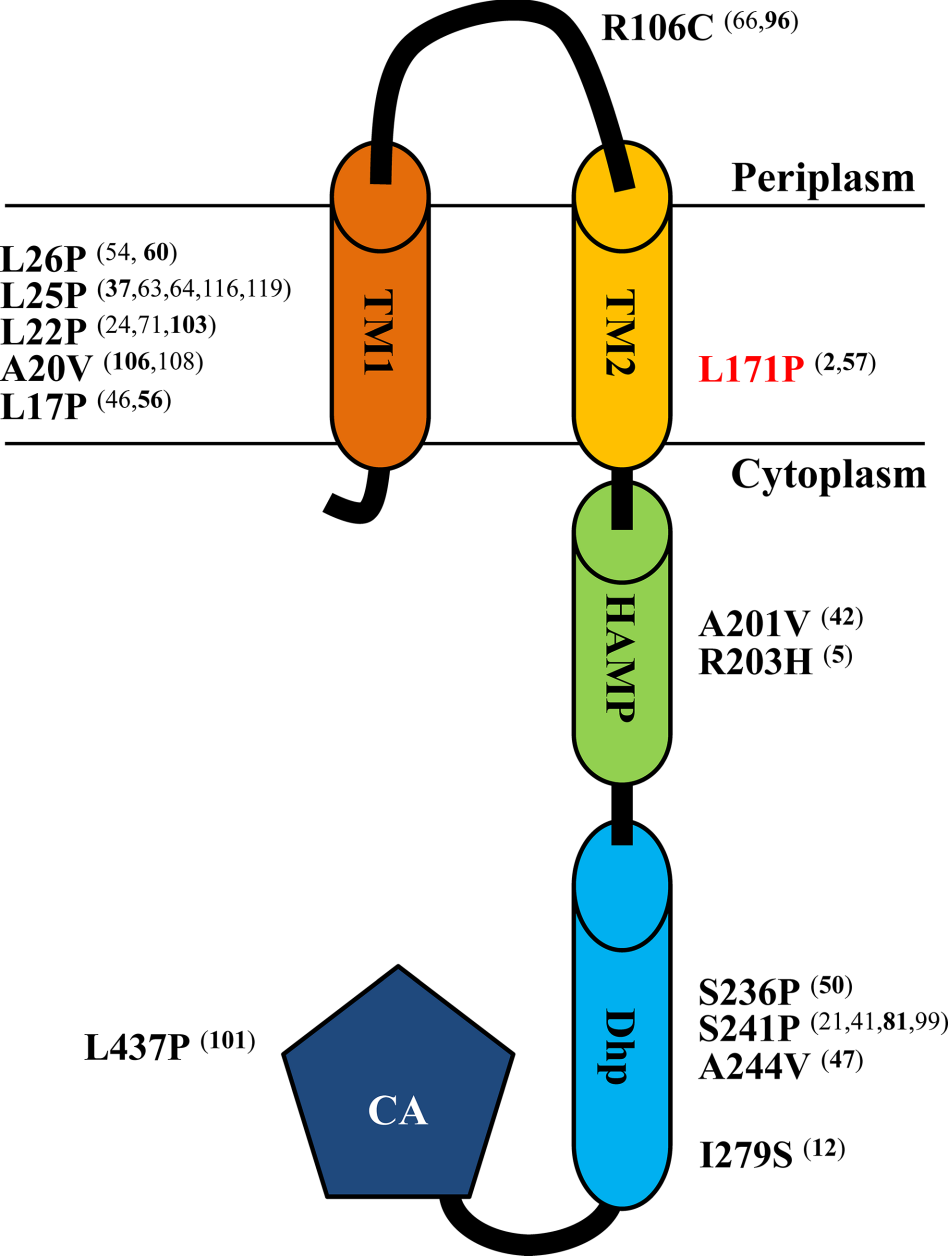
Positions of the single amino acid substitutions on CpxS leading to reduced antibiotic susceptibility in *P. aeruginosa*. Numbers in the superscript parentheses are the isolated strain numbers (Table S2) containing the corresponding substitution as either a single substitution (in bold) or one of multiple substitutions (in plain). The amino acid substitutions that appeared in published genomes of *P. aeruginosa* isolates are marked in red. C-terminal catalytic and ATP-binding domain.

In order to investigate if any other *cpxS* mutation can lead to reduced antibiotic susceptibility, we conducted random mutagenesis screening of *cpxS* in *P. aeruginosa*. The plasmids harboring mutated *cpxS* were constructed by error-prone PCR and introduced into PA14Δ*cpxS*, and the transformants were screened for reduced susceptibility to ciprofloxacin (MIC ≥2-fold). A total of 83 different CpxS mutants were confirmed to be associated with reduced antibiotic susceptibility by re-introducing the purified plasmid harboring each mutated *cpxS* gene into PA14Δ*cpxS* (Table S2). Among them, 14 non-redundant CpxS mutants had single amino acid substitution (Fig. 1), including Leu26Pro, as in the case of PA14OCR109. Introduction of the plasmid harboring each of these 14 CpxS mutants led to reduced susceptibility to fluoroquinolones and β-lactams in PA14Δ*cpxS* (Table 1). Next, it was investigated if occurrence of corresponding mutations in the chromosomal *cpxS* could exert a similar effect. In this case, three single amino acid substitutions (Leu26Pro, Ser236Pro, and Ser241Pro) were chosen to introduce into the PA14 chromosome, as each of these amino acid substitutions could be obtained by single nucleotide substitution (^76^CTG → CCG, ^706^TCC → CCC, and ^721^TCG → CCG, respectively). When each of these single nucleotide substitutions was introduced into the chromosome through double cross-over recombination, reduced antibiotic susceptibility was observed in *P. aeruginosa* PA14 (Table 1). These results demonstrated that a single nucleotide substitution in the chromosomal *cpxS* locus could result in reduced antibiotic susceptibility in *P. aeruginosa* PA14. These results also demonstrated that the single nucleotide mutation in the *cpxS* locus was sufficient to cause reduced antibiotic susceptibility in the PA14OCR109 isolate mentioned above. Taken together, our work unraveled the existence of *cpxS* mutational resistome in *P. aeruginosa*.

In order to investigate the presence of relevant *cpxS* mutations in the published *P. aeruginosa* genomes, we searched the *cpxS* coding regions of 3,999 published genomes of *P. aeruginosa* isolates by BLASTN in the Pseudomonas Genome Database (23). A total of 269 genomes harbor exactly the same nucleotide sequence of the *cpxS* coding region as the PA14 strain, while others harbor 1 up to 24 nucleotide variations (Table S3). The amino acid substitutions identified in these genomes were located on various domains of the CpxS protein, and among them, four amino acid substitutions appeared in the CpxS mutants identified in this work (Fig. S2). Notably, the Leu171Pro single amino acid substitution occurred in the *cpxS* coding regions of two *P. aeruginosa* clinical isolates RNS_PAE05 and RNS_PA46 (Fig. S3), which have shown extensive antibiotic resistance (24). These results indicate that these *cpxS* mutations have clinical relevance.

### The reduced antibiotic susceptibility phenotype of the *cpxS* mutant strains is *cpxR* dependent

**FIG 2:**
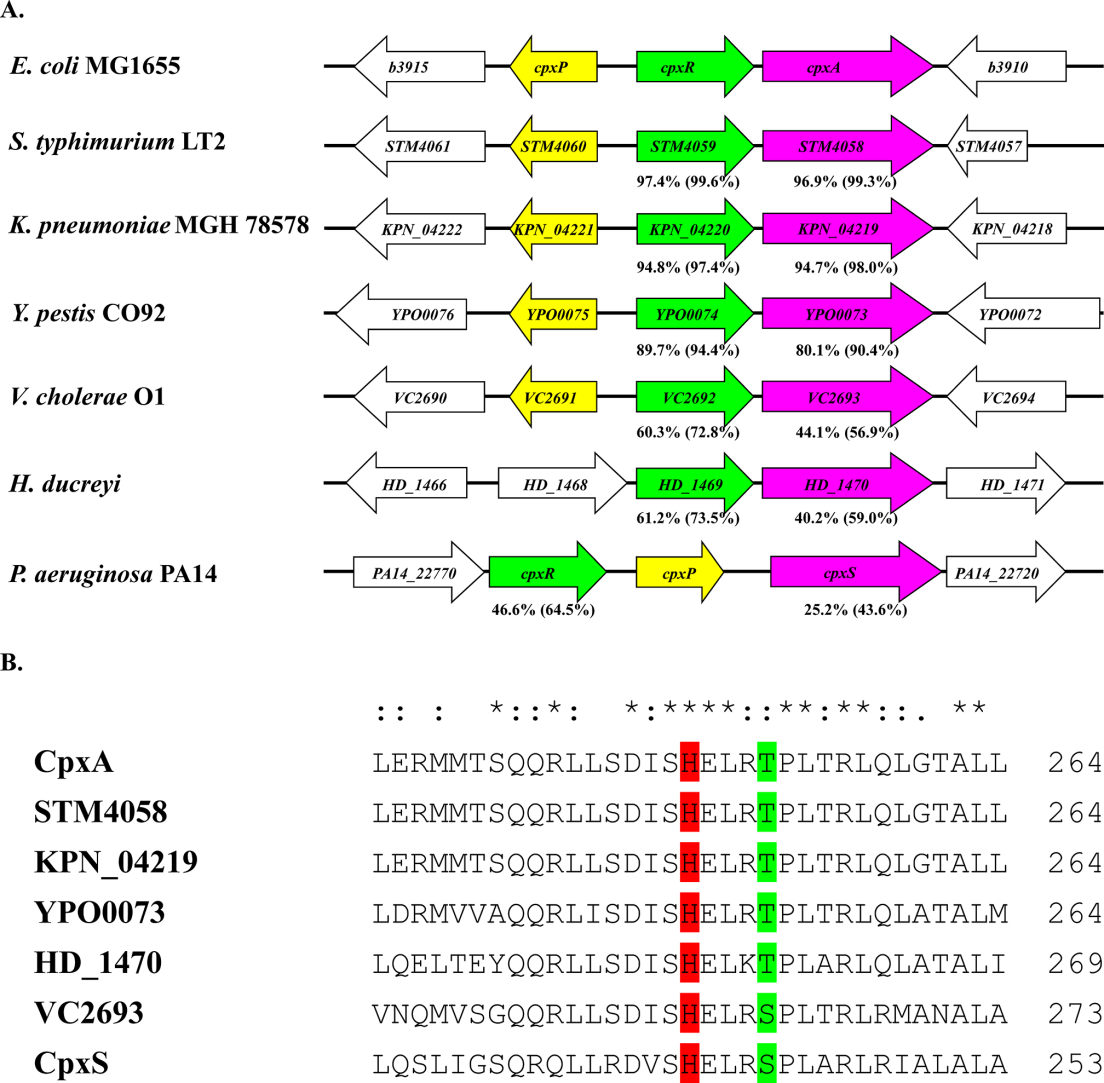
Comparison of the gene arrangement of the *cpx* loci among different bacterial species (**A**). Arrows indicate the orientation of each gene, and ortholog genes are marked in the same color. Comparison of amino acid sequences around the autophosphorylation histidine residue among the CpxA orthologs (**B**). The fourth residue (marked in green) downstream of the autophosphorylation histidine residue (marked in red) is a key determinant for the enzyme activity in EnvZ family sensor kinase.

In the genome of *P. aeruginosa* PA14, *cpxS* is a gene coding for a putative sensor kinase, and the nearest gene coding for a response regulator is *cpxR*, located 683-bp upstream of *cpxS*. The *cpxP* locus, a target gene of CpxR encoding a small periplasmic protein with LTXXQ motifs (14), is located between *cpxR* and *cpxS* with the same orientation (Fig. 2A). In the genomes of many bacteria, *cpxR* and its cognate sensor kinase gene *cpxA*, as well as its target gene, *cpxP*, comprise the *cpx* loci. Using KEGG genome database, we compared the organization of the *cpx* loci among bacterial genomes. In many bacteria such as *Escherichia coli*, *Salmonella typhimurium*, *Klebsiella pneumoniae*, *Yersinia pestis*, and *Vibrio cholerae*, *cpxP* is oriented divergently from the *cpxRA* operon. Apparently, gene arrangement of the *cpx* loci is quite different in the genome of the *P. aeruginosa* PA14 (Fig. 2A). Although the alignment shows relatively low identity (25.2%, similarity 43.6%) in the primary sequences, the overall feature of the predicted domain structure of CpxS protein is quite similar to that of *E. coli* CpxA protein (Fig. S1). Particularly, the CpxS protein has a highly conserved motif around the autophosphorylation histidine residue along with CpxA proteins from other bacteria belonging to the EnvZ family sensor kinases (Fig. 2B).

Since *cpxR* is located near *cpxS* in the genome of *P. aeruginosa*, we investigated whether *cpxR* was involved in the reduction of antibiotic susceptibility in the above *cpxS* mutant strains. Deletions of *cpxR* abolished the elevation of the MICs of antibiotics in these genetic backgrounds (Table 1). Although *cpxS* unlikely comprise an operon with *cpxR* in the genome of *P. aeruginosa*, as *cpxP* is located in between of *cpxR* and *cpxS* (Fig. 2A) and CpxR can bind the promoter and activate the expression of *cpxP* (14), a possible polar effect of *cpxR* deletion was concerned and ruled out by introducing the plasmid harboring the mutant *cpxS* (Table 1). These results demonstrated that the reduced antibiotic susceptibility in these *cpxS* mutant strains was dependent on *cpxR*.

### Increased expression of the *mexAB-oprM* operon in the *cpxS* mutants

**FIG 3:**
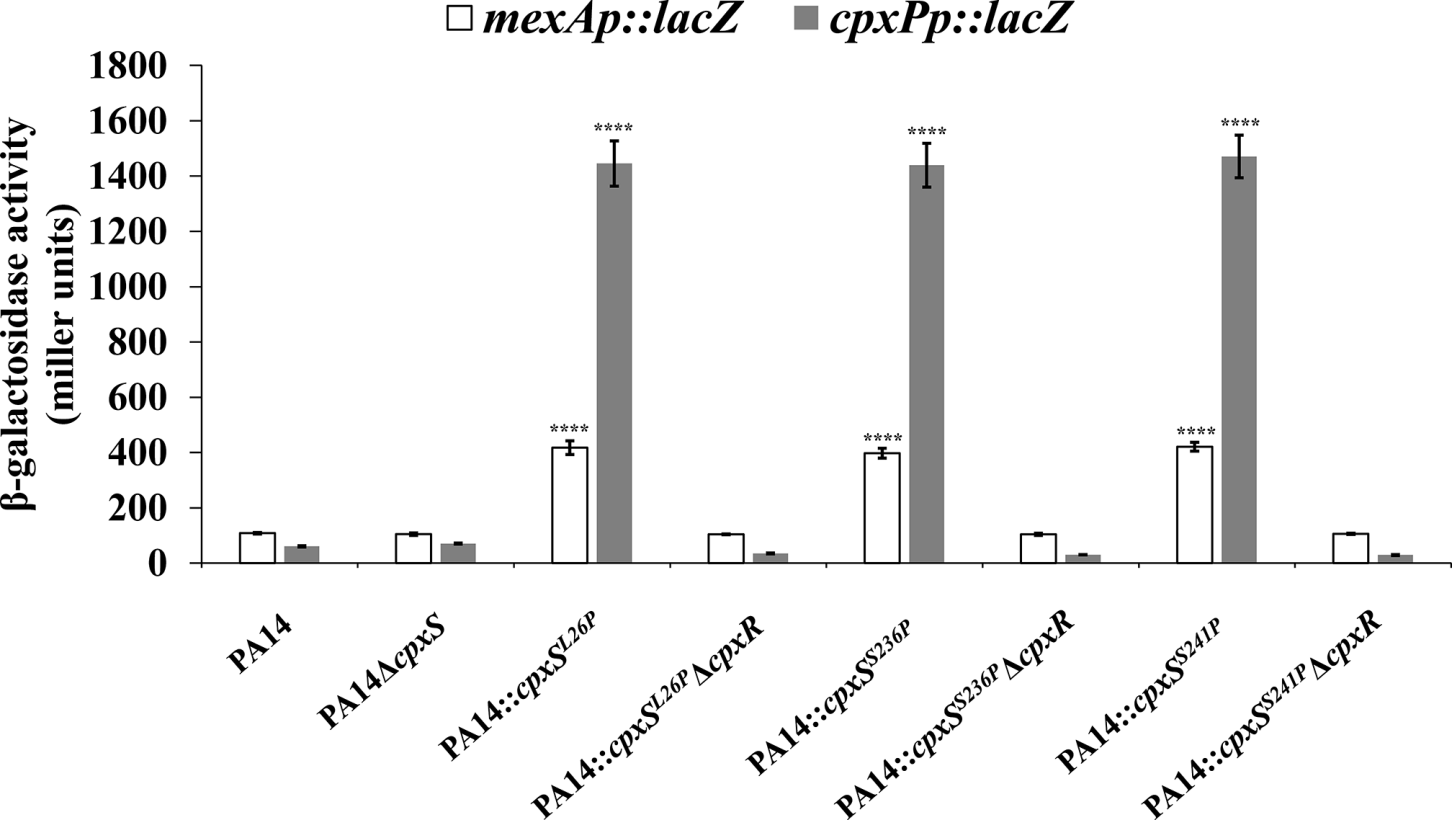
The expression levels of *mexA* and *cpxP* are increased in the *cpxS* mutated *P. aeruginosa* strains in a *cpxR-*dependent manner. Cells containing reporter plasmids were grown to exponential phase in Mueller-Hinton broth at 37°C. The results are presented as the mean values from triplicate samples. Statistical analysis was performed using an unpaired two-tailed Student *t*-test. ****P value was lower than 0.0001, when the control strain was set as either PA14 or PA14Δ*cpxS* or each of the *cpxR* deleted strains.

The *mexAB-oprM* operon, coding for the predominant multidrug efflux pump, is a member of the CpxR regulon, as CpxR can bind and activate the promoter of the *mexAB-oprM* operon in *P. aeruginosa* (14). Since CpxR mediates the reduced antibiotic susceptibility in the above *cpxS* mutant strains, it was rational to investigate whether the expression level of the *mexAB-oprM* operon was increased in these *cpxS* mutant strains. A *mexA*p::*lacZ* reporter plasmid (14) was introduced into these strains. The expression level of *mexA*p::*lacZ* was increased in these *cpxS* mutant strains (Fig. 3). When the *mexA*p::*lacZ* reporter plasmid was introduced into the *cpxR* deleted version of these strains, the increase was abolished (Fig. 3). These results demonstrated that these *cpxS* mutant *P. aeruginosa* strains had increased expression level of the *mexAB-oprM* operon in a *cpxR*-dependent manner.

Previously, we identified a new *nalB*-type *P. aeruginosa* isolate (PA14OCR36) showing increased expression level of the *mexAB-oprM* operon in a *cpxR*-dependent manner (14). This isolate also showed highly increased expression of *cpxP*, the cognate target gene of CpxR. When the *cpxP*p::*lacZ* reporter plasmid (14) was introduced into the above *cpxS* mutant strains, significant induction of *cpxP* expression was observed (Fig. 3), which showed a similar pattern with PA14OCR36 (14). However, different mechanisms should be involved since no mutation occurred in the *cpxS* gene in PA14OCR36.

We further investigated the contribution of the MexAB-OprM pump to the reduced antibiotic susceptibility in these *cpxS* mutant strains by deleting *mexA* gene in each strain. These *mexA* deleted strains had the same low levels of MICs of antibiotics as PA14Δ*mexA*, showing a drastic increase of antibiotic susceptibility compared to their parental strains (Table 1). These results indicated that *mexA* is prerequisite for the reduced antibiotic susceptibility in these *cpxS* mutant strains.

## DISCUSSION

In this work, we have demonstrated that at least 14 single amino acid mutations in CpxS can lead to reduced antibiotic susceptibility in *P. aeruginosa* PA14 (Table 1). By comparing the occurrences of amino acid substitutions among the CpxS variants with multiple mutations (Table S2), additional candidates could be selected, such as Ala211Val, Leu252Pro, Asn215Asp, and Leu18Pro, which appeared in more than two different isolates, and need further confirmation. Anyway, *cpxS* is a novel member of mutational resistome in *P. aeruginosa*. Previous resistome analyses were based on insertion mutagenesis (2 – 7); apparently, the *cpxS* locus could not be screened out in these cases. The *cpxS* mutant strains showing reduced antibiotic susceptibility could be isolated *in vitro* from the treatment of certain antibiotics in *P. aeruginosa* (Table 1). Several *P. aeruginosa* isolates have amino acid mutations identified in this work in its *cpxS* coding region (Fig. S3), showing clinical relevance of the *cpxS* mutational resistome.

Among the single amino acid substitutions of CpxS, the Ser241Pro substitution is particularly notable, as it is located in the highly conserved region of EnvZ family sensor kinases (Fig. 2B). This serine residue is located at four residues downstream of the conserved autophosphorylation histidine residue in *P. aeruginosa* CpxS and *V. cholerae* CpxA, while a threonine residue is located at four residues downstream of the conserved autophosphorylation histidine residue in other bacterial CpxA (marked green in Fig. 2B). It was reported that this theronine residue was a key determinant for modulating the enzyme activities in the EnvZ family sensor kinases (25). Alterations of this threonine residue caused versatile signaling output; for example, substitution to tyrosine caused high constitutive kinase activity; substitution to glutamic acid abolished kinase activity, while substitution to serine caused no changes to the functions of the sensor kinase (25). When the threonine residue was substituted to proline, *E. coli* CpxA revealed high constitutive kinase activity (26). We propose that Ser241Pro substitution results in constitutively active CpxS in *P. aeruginosa*. Furthermore, numerous constitutively active CpxA mutants were isolated, and the locations of these mutants scattered on the different regions of the protein in *E. coli* (18, 26, 27). Thus, we further propose that these CpxS mutants identified in this work are indeed constitutively active mutants, and CpxS, together with CpxR, comprises a two-component regulatory system in *P. aeruginosa*.

The positions of these single amino acid substitutions leading to reduced antibiotic susceptibility were distributed to various functional domains of the CpxS protein (Fig. 1). Thus, various mechanisms may be engaged by these mutants to cause CpxR activation, namely, signal perception, conformational change transmission, autophosphorylation, kinase activity, as well as phosphatase activity.

In this work, we have demonstrated that CpxS is linked to the regulation of the MexAB-OprM efflux pump expression in *P. aeruginosa* in a CpxR-dependent manner (Fig. 3). Previously, we demonstrated that CpxR was directly involved in the regulation of the expression of two resistance-nodulation-cell division (RND) efflux pumps, MexAB-OprM and MuxABC-OpmB, in *P. aeruginosa* (14). The expression of MexAB-OprM, but not MuxABC-OpmB, activated by CpxR is responsible for the reduced antibiotic susceptibility. Furthermore, the regulatory effect of CpxR on the expression of MexAB-OprM is specific to *P. aeruginosa* strains, while the regulatory effect of CpxR on the expression of MuxABC-OpmB is common among different *Pseudomonas* species (14). The gene arrangement of the *cpx* loci is similar among *Pseudomonas* species (see Fig. S4), which is different from other bacterial species, such as *E. coli* and *V. cholerae* (Fig. 2A). It is notable that the regulatory role of the CpxS-CpxR two-component regulatory system on the expression of MexAB-OprM is specific to *P. aeruginosa*, one of the notorious pathogens prone to develop multidrug resistance.

Here we propose a model to depict the possible contribution of the CpxS-CpxR two-component regulatory system to multidrug resistance in *P. aeruginosa* (Fig. S5). In three circumstances, CpxS-CpxR may modulate the expression of *mexAB-oprM*. Firstly, the emergence of the *cpxS* mutational resistome was identified in this work, likely due to the constitutively active mutations, which may bypass signals. Secondly, mutational resistome genes other than *cpxS*, but dependent on *cpxS-cpxR*, should exist in *P. aeruginosa*. Notably, PA14OCR36, a strain with elevated expression levels of *mexAB-oprM* as well as *cpxP*, but with intact *cpxS*, was isolated under the same conditions as the *cpxS* mutated PA14OCR109 (14). As the Cpx system is involved in sensing and responding to misfolded *P* pilin proteins caused by gene mutation in *E. coli* (28), it is rational to propose that the wild-type CpxS proteins may sense stress generated by certain misfolding envelope proteins in *P. aeruginosa*. Thirdly, the wild-type CpxS proteins may also sense cell envelope stress generated by certain extracellular physical/chemical perturbations in *P. aeruginosa*, since in many Gram-negative bacteria, the Cpx system is involved in cell envelope protection against various stresses such as pH, salinity, and heavy metal (15 – 19). Then CpxS phosphorylates CpxR, which in turn activates CpxR regulon genes, such as *cpxP*, *muxABC-opmB*, and *mexAB-oprM* (14). MexAB-OprM efflux pump upregulated by CpxS-CpxR might contribute to maintain the cellular homeostasis challenged by the cell envelope stresses, since it is emerging that multidrug efflux pumps have physiological roles rather than antibiotic resistance (29). In this case, the CpxS-CpxR two-component regulatory system contributes to multidrug resistance as a cellular protection process in *P. aeruginosa*. The characterization of the innate signals sensed by CpxS is undergoing.

## Data Availability

The whole genome sequencing data can be found in the NCBI database (accession No. PRJNA895925).

## SUPPLEMENTAL MATERIAL

Fig. S1 aac.00921-23-s0001.pdf Comparison of primary amino acid sequences and secondary structures of *P. aeruginosa* CpxS protein and *E. coli* CpxA protein

Fig. S2 aac.00921-23-s0002.pdf Positions of the amino acid substitutions of CpxS in the published genomes of *P. aeruginosa* isolates

Fig. S3 aac.00921-23-s0003.pdf Nucleotide sequence alignment of the *cpxS* coding regions of RNS_PAE05, RNA_PA46 and PA14

Fig. S4 aac.00921-23-s0004.pdf Comparison of the gene arrangement of the *cpx* loci among different *Pseudomonas* species

Fig. S5 aac.00921-23-s0005.pdf The proposed model depicting the contributions of the CpxS-CpxR two component regulatory system to multidrug resistance in *P. aeruginosa*

Table S1 aac.00921-23-s0006.pdf Sequences of DNA primers used in this study

Table S2 aac.00921-23-s0007.pdf List of the CpxS mutants, isolated from random mutagenesis generated by error-prone PCR, leading to reduced susceptibility to ciprofloxacin

Table S3 aac.00921-23-s0008.xls BlastN alignment of the *cpxS* coding region of PA14 with the *cpxS* coding regions of 3999 published genomes of *P. aeruginosa* isolates

ASM does not own the copyrights to Supplemental Material that may be linked to, or accessed through, an article. The authors have granted ASM a non-exclusive, world-wide license to publish the Supplemental Material files. Please contact the corresponding author directly for reuse.
