# Supplementary material for: Identification of cpxS mutational resistome in Pseudomonas aeruginosa
Source: Antimicrob Agents Chemother. 2023 Oct 6;67(11):e00921-23. doi: 10.1128/aac.00921-23 (PMC10648845; doi:10.1128/aac.00921-23)
Supplement: Fig. S1 — Comparison of primary amino acid sequences and secondary structures of P. aeruginosa CpxS protein and E. coli CpxA protein [file aac.00921-23-s0001.pdf]

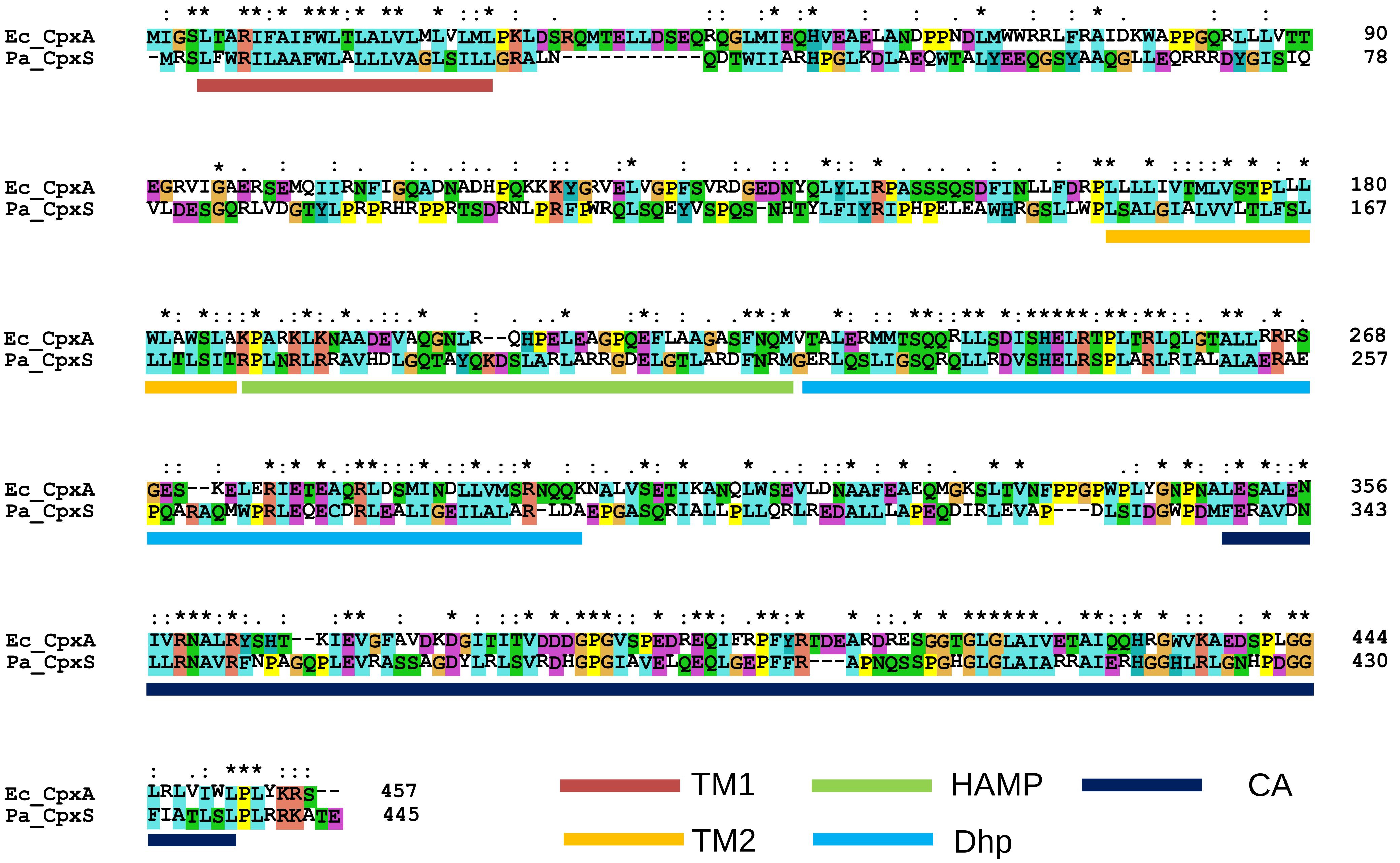

Fig. S1. Comparison of primary amino acid sequences and secondary structures of *P. aeruginosa* CpxS protein and *E. coli* CpxA protein. Primary amino acid sequences were aligned using ClustalX2 software. TM1, first transmembrane region; TM2, second transmembrane region; HAMP, HAMP signaling domain; Dhp, dimerization and histidine phosphotransfer domain; CA, catalytic and ATP-binding domain.
