# Supplementary material for: Identification of cpxS mutational resistome in Pseudomonas aeruginosa
Source: Antimicrob Agents Chemother. 2023 Oct 6;67(11):e00921-23. doi: 10.1128/aac.00921-23 (PMC10648845; doi:10.1128/aac.00921-23)
Supplement: Fig. S2 — Positions of the amino acid substitutions of CpxS in the published genomes of P. aeruginosa isolates [file aac.00921-23-s0002.pdf]

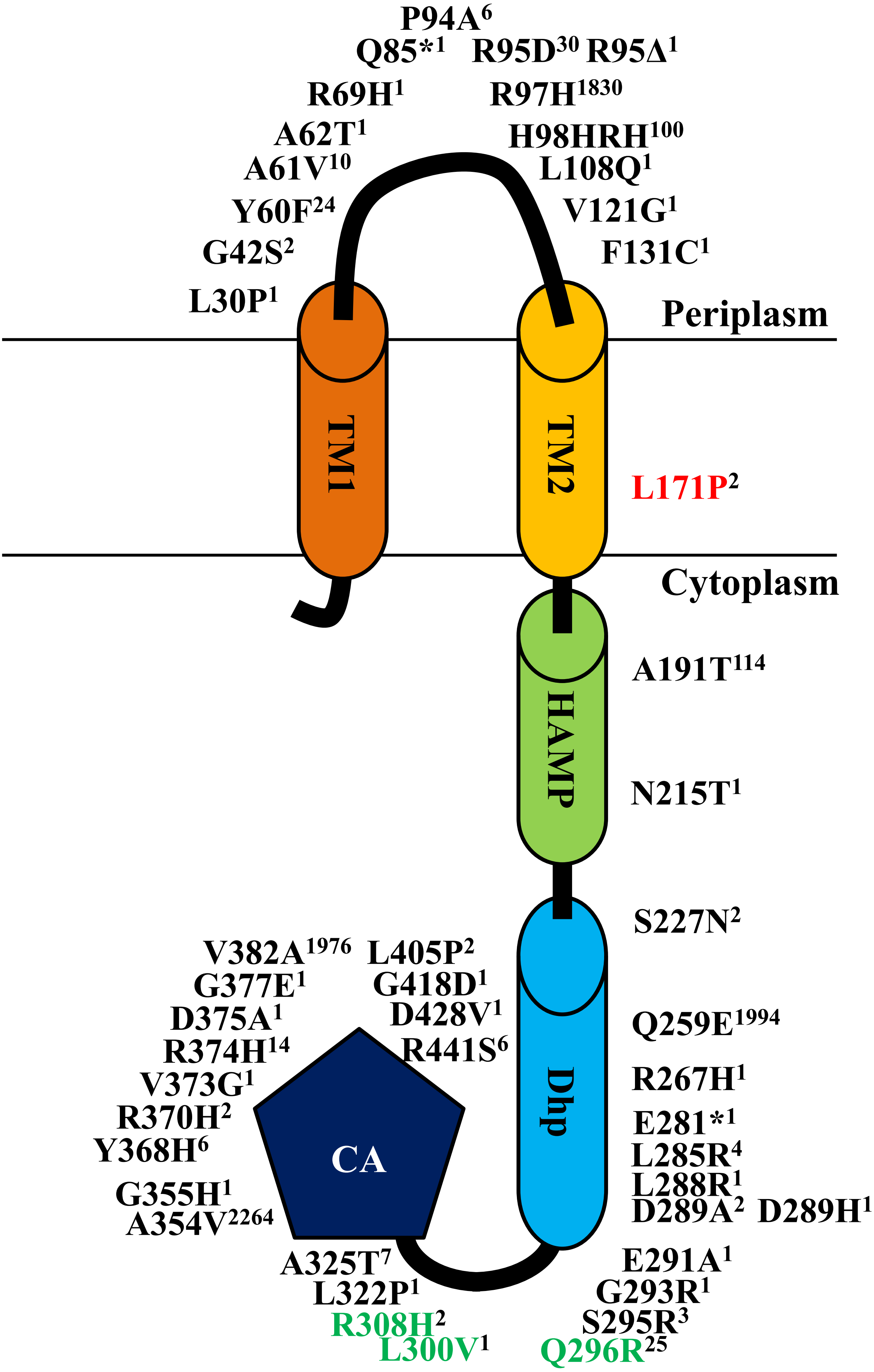

Fig. S2. Positions of the amino acid substitutions of CpxS in the 3999 published genomes of *P. aeruginosa* isolates (Supplementary Table S3). Numbers in the superscript are the occurrence times in different genomes. The amino acid substitution marked in red appeared in the single substitution, while those marked in green appeared in the multiple substitution (Supplementary Table S2).
