# Supplementary material for: Identification of cpxS mutational resistome in Pseudomonas aeruginosa
Source: Antimicrob Agents Chemother. 2023 Oct 6;67(11):e00921-23. doi: 10.1128/aac.00921-23 (PMC10648845; doi:10.1128/aac.00921-23)
Supplement: Fig. S3 — Nucleotide sequence alignment of the cpxS coding regions of RNS_PAE05, RNA_PA46 and PA14 [file aac.00921-23-s0003.pdf]

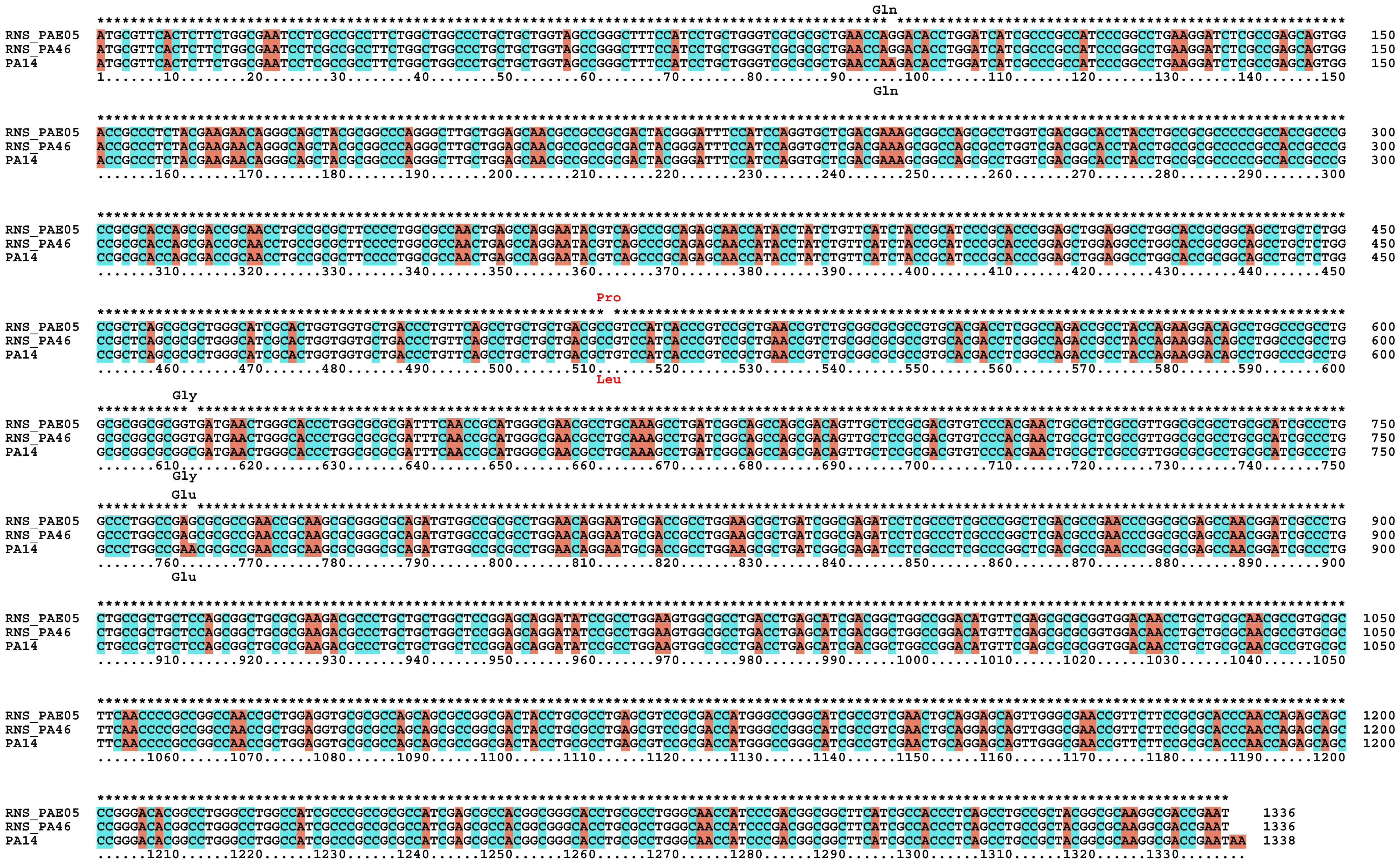

Fig. S3. Nucleotide sequence alignment of the *cpxS* coding regions of RNS_PAE05, RNA_PA46 and PA14. Three synonymous mutations and one missense mutation (Leu171Pro) were identified in RNS_PAE05 and RNA_PA46 compared to PA14.
