# Supplementary material for: Identification of cpxS mutational resistome in Pseudomonas aeruginosa
Source: Antimicrob Agents Chemother. 2023 Oct 6;67(11):e00921-23. doi: 10.1128/aac.00921-23 (PMC10648845; doi:10.1128/aac.00921-23)
Supplement: Fig. S4 — Comparison of the gene arrangement of the cpx loci among different Pseudomonas species [file aac.00921-23-s0004.pdf]

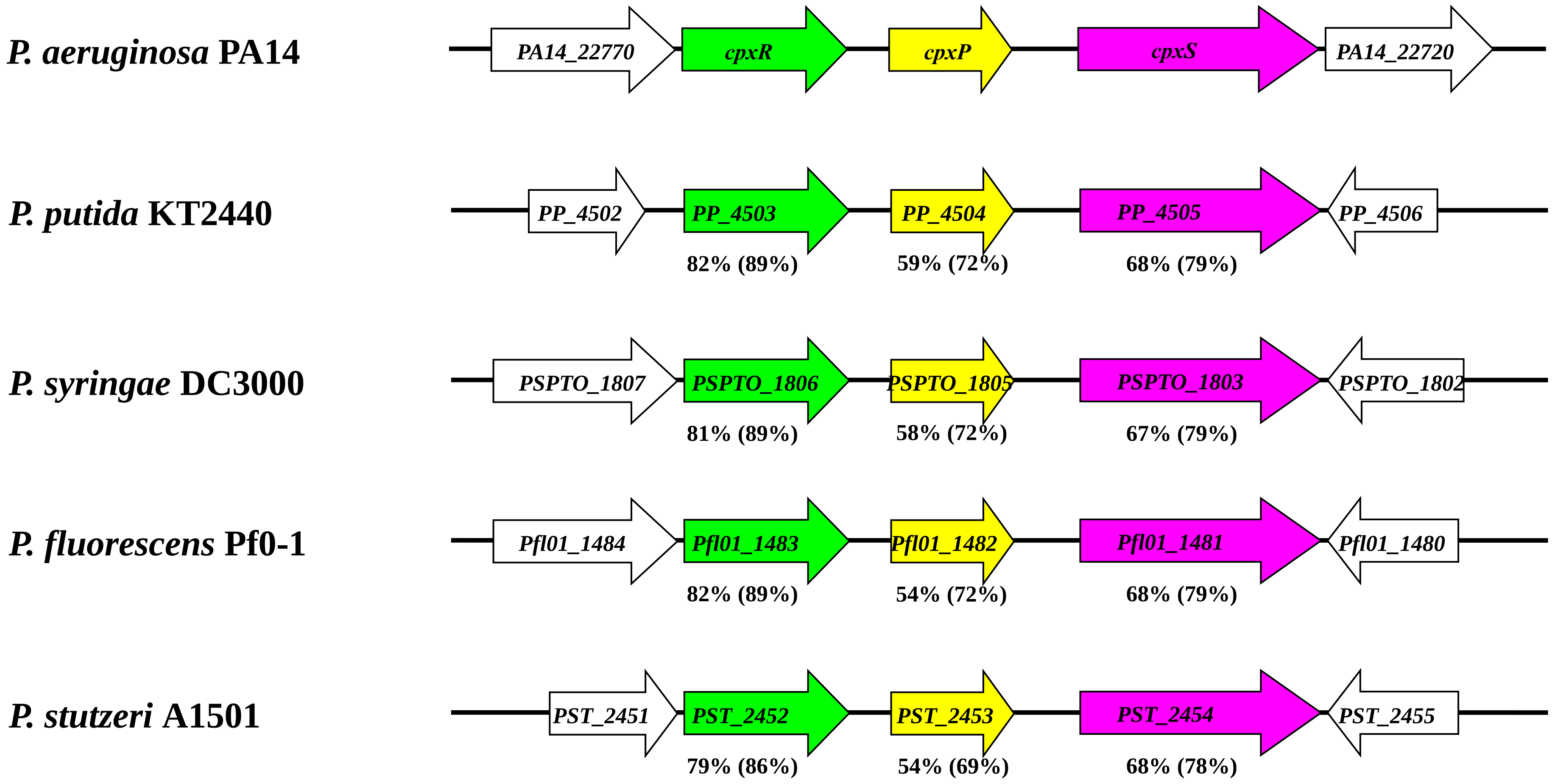

Fig. S4. Comparison of the gene arrangement of the cpx loci among different *Pseudomonas* species. Arrow indicates the orientation of each gene and orthologues genes are marked in the same colour.
