# Supplementary material for: Identification of cpxS mutational resistome in Pseudomonas aeruginosa
Source: Antimicrob Agents Chemother. 2023 Oct 6;67(11):e00921-23. doi: 10.1128/aac.00921-23 (PMC10648845; doi:10.1128/aac.00921-23)
Supplement: Fig. S5 — The proposed model depicting the contributions of the CpxS-CpxR two component regulatory system to multidrug resistance in P. aeruginosa [file aac.00921-23-s0005.pdf]

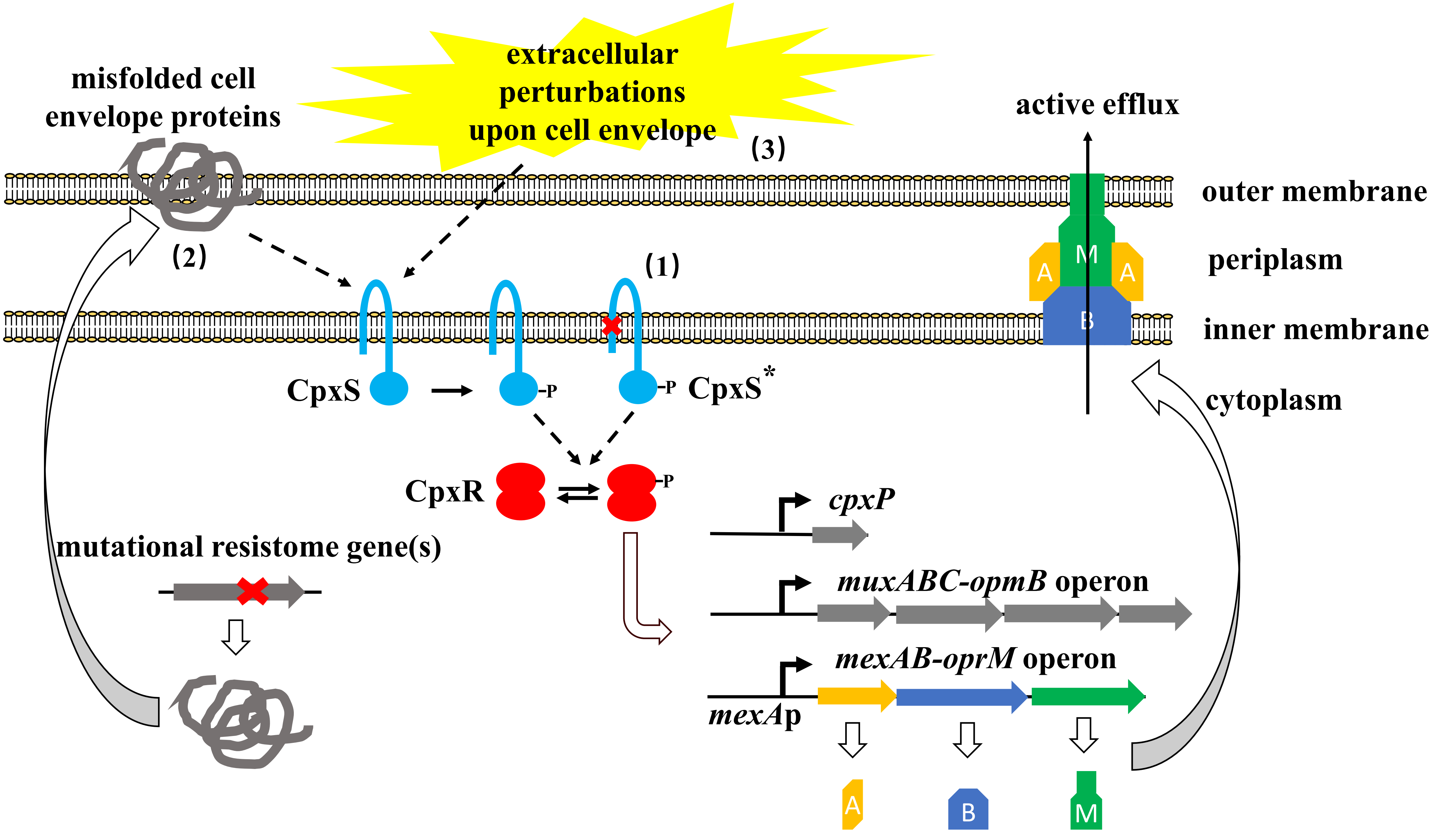

Fig. S5. The proposed model depicting the contributions of the CpxS-CpxR two component regulatory system to multidrug resistance in *P. aeruginosa*. (1) CpxS*, putative constitutively active mutants, identified in this work; (2) misfolded envelope proteins encoded by mutated resistome genes, inspired from the Cpx signaling in *E. coli* (ref. 28); (3) extracellular perturbations upon cell envelope such as pH, salinity, heavy metal *etc*., inspired from the Cpx signaling in many bacteria (refs. 15-19). Dashed arrows denote predicted effects requiring further verification.
