# Supplementary material for: Identification of cpxS mutational resistome in Pseudomonas aeruginosa
Source: Antimicrob Agents Chemother. 2023 Oct 6;67(11):e00921-23. doi: 10.1128/aac.00921-23 (PMC10648845; doi:10.1128/aac.00921-23)
Supplement: Table S1 — Sequences of DNA primers used in this study [file aac.00921-23-s0006.pdf]

Supplementary Table S1. Sequences of DNA primers used in this study

	5′→3′
**Primers for locus deletion**	
cpxS upstream region F primer (*Hind*III)#	CCCAAGCTTTACCCAGGGCTGAC
R primer (*Sal*I)	ACGCGTCGACATGGTTTTCCTGTTGAATCGC
cpxS downstream region F primer (*Sal*I)	ACGCGTCGACGCAAGGCGACCGAATGACGC
R primer (*Bam*HI)	GGGGATCCGGCTTCGTCTACCTGGGTACG
cpxR upstream region F primer (*Bam*HI)	GGGGATCCCGGCAAACGTTTTCGCACC
R primer (*Kpn*I)	CAGGTACCGAGCAGCTCGCAGAG
cpxR downstream region F primer (*Kpn*I)	GGGGTACCTGCGCAAGAAGCTCGGCAGC
R primer (*Eco*RI)	GGAATTCGGCCCACTCTGCCTTGC
mexA upstream region F primer (*Xba*I)	GCTCTAGACATTGTGCTTCGAAAAGAATGTTC
R primer (*Bam*HI)	CGGGATCCGGTTTACTCGGCCAAACCAATG
mexA downstream region F primer ((*Bam*HI)	CGGGATCCGCTACACCAAGGTGCTGTCGC
R primer (*Kpn*I)	GGGGTACCTTCGGTGACCAGCCACTTGTCG
**Primers for pUC18 derived *cpxS* template plasmid construction**	
F primer (*Eco*RI)	GGAATTCGCATATCCTAGAGGTTTACC
R primer (*Bam*HI)	GGGATCCGTTCATCGAGGAACAACGAAGC
**Primers for nest-PCR introducing mutant *cpxS***	
Leu26Pro F primer	AGCCGGGCTTTCCATCCTGCCGGGTCGCGCGCTGAACCAAG
R primer	CTTGGTTCAGCGCGCGACCCGGCAGGATGGAAAGCCCGGCT
Ser236Pro F primer	ACAGTTGCTCCGCGACGTGCCCCACGAACTGCGCTCGCC
R primer	GGCGAGCGCAGTTCGTGGGGCACGTCGCGGAGCAACTGT
Ser241Pro F primer	GTGTCCCACGAACTGCGCCCGCCGTTGGCGCGCCTGC
R primer	GCAGGCGCGCCAACGGCGGGCGCAGTTCGTGGGACAC

Restriction sites are underlined in the sequences of primers.
