# Supplementary material for: Identification of cpxS mutational resistome in Pseudomonas aeruginosa
Source: Antimicrob Agents Chemother. 2023 Oct 6;67(11):e00921-23. doi: 10.1128/aac.00921-23 (PMC10648845; doi:10.1128/aac.00921-23)
Supplement: Table S2 — List of the CpxS mutants, isolated from random mutagenesis generated by error-prone PCR, leading to reduced susceptibility to ciprofloxacin [file aac.00921-23-s0007.pdf]

Supplementary Table S2. List of the CpxS mutants, isolated from random mutagenesis generated by error-prone PCR, leading to reduced susceptibility to ciprofloxacin (MIC≥2-fold).

Isolate No.	Mutated residue(s)
1	G58D, F392S
2*	L171P
3	T34A, R134H, L268P
4	L200P, P396L
5	R203H
8	R134H, I157V, A408T
9	E48K, Q228L, Q270R
12	I279S
13	R86C, L177P, L300V, F351S
14	L43P, L252P#
15	L167P, I173V, P242S, D273G
17	L149P, Q259R
18	L108P, L162P, R308H, W333C, S363C
21	H144R, T209A, S241P
22	L252P, G280D, A290T, I380V, R441H
23	I132T, K194R, R233C, A395V
24	L22P, L268Q
27	E141G, L252P, I380T
30	S227G, E254K, M336V
33	N215D, H376R
34	I77T, R216C, L250P, S363N, I414V
35	I36N, L87P, A244E, A262V
36	R412H, P427L
37	L25P
39	L200P, G293D
41	S241P, G383V
42	A201V
43	Q263R, E291G, G424D
44	E254G, L285P, L300P
45	R39P, R361C, Q385R
46	L17P, W35R, Q78L, F131L, N39D, T444A
47	A244V
48	G156S, L300Q, A301V
49	Q193R, A395T, K442R
50	S236P
51	Q63L, F111I, Q118R, A211V
52	D72G, L80P
53	S23P, A362V
54	L26P, Q318L, S364N
55	P138L, F392S
56	L17P
57	L171P
58	L16P, R102H, Y120H, A365V
59	L155P, L283P
60	L26P
62	V121A, L177P, L384Q

63	L25P, L197P
64	A20T, L25P, L30R, F351S
65	I248T, G389D
66	R106C, H144Y, Q193R
68	Y60H, N215D, L283P, D342G
71	L22P, L108P
74	L18P, I36T, M336T
75	G84D, R106H, L252Q, A260G, W333R
76	L18P, L30P, T51A
79	L288F, P302F, L303Q, M336I
80	D33G, A211V
81	S241P
83	P94L, R97C, R99C, Y368C
84	A191V, L231S
85	S23F, Δ(P96-R110), I432S
86	G208D, A260V, V349M
87	N215D, V324A, R411H
89	L80P, R199H
91	G156D, R287P
95	L164P, A211V, D327G
96	R106C
98	A211V, I298V
99	R110C, R199H, S241P, Q296R
100	Q193L, L232F, R274C, L288P, F337L
101	L437P
102	N31D, L140P, D195G, E383K
103	L22P
104	I132V, Y133S, L197P
105	P109L, R114C, R145C
106	A20V
108	A20V, F431L
109	N126D, L285H, Q398L
111	L18P, G429D, A433V
112	S3P, P136L, L177R, R240C
115	R114C, D367Y
116	L25P, V235A
117	K44E, Q68R, A251T, H417L
119	L25P, Y120H, E317G, A340T

* Isolate with single amino acid substitution was marked in red.

# Amino acid substitution appeared in more than 2 isolates with multiple substitutions were highlighted by the same background color.
